# To have value, comparisons of high-throughput phenotyping methods need statistical tests of bias and variance

**DOI:** 10.3389/fpls.2023.1325221

**Published:** 2024-01-19

**Authors:** Justin M. McGrath, Matthew H. Siebers, Peng Fu, Stephen P. Long, Carl J. Bernacchi

**Affiliations:** ^1^ Global Change and Photosynthesis Research Unit, USDA-Agricultural Research Service (ARS), Urbana, IL, United States; ^2^ Department of Plant Biology, University of Illinois, Urbana-Champaign, Urbana, IL, United States; ^3^ Center for Advanced Agriculture and Sustainability, Harrisburg University of Science and Technology, Harrisburg, PA, United States; ^4^ Carl R. Woese Institute for Genomic Biology, University of Illinois, Urbana-Champaign, Urbana, IL, United States; ^5^ Department of Crop Sciences, University of Illinois, Urbana-Champaign, Urbana, IL, United States

**Keywords:** physical sciences, statistics method comparison, variance, bias, limits of agreement, Bland and Altman

## Abstract

The gap between genomics and phenomics is narrowing. The rate at which it is narrowing, however, is being slowed by improper statistical comparison of methods. Quantification using Pearson’s correlation coefficient (*r*) is commonly used to assess method quality, but it is an often misleading statistic for this purpose as it is unable to provide information about the relative quality of two methods. Using *r* can both erroneously discount methods that are inherently more precise and validate methods that are less accurate. These errors occur because of logical flaws inherent in the use of *r* when comparing methods, not as a problem of limited sample size or the unavoidable possibility of a type I error. A popular alternative to using *r* is to measure the limits of agreement (LOA). However both *r* and LOA fail to identify which instrument is more or less variable than the other and can lead to incorrect conclusions about method quality. An alternative approach, comparing variances of methods, requires repeated measurements of the same subject, but avoids incorrect conclusions. Variance comparison is arguably the most important component of method validation and, thus, when repeated measurements are possible, variance comparison provides considerable value to these studies. Statistical tests to compare variances presented here are well established, easy to interpret and ubiquitously available. The widespread use of *r* has potentially led to numerous incorrect conclusions about method quality, hampering development, and the approach described here would be useful to advance high throughput phenotyping methods but can also extend into any branch of science. The adoption of the statistical techniques outlined in this paper will help speed the adoption of new high throughput phenotyping techniques by indicating when one should reject a new method, outright replace an old method or conditionally use a new method.

## 1 Introduction

Advancements in sequencing technologies have created a gap in our understanding of the relationship between genetic vs phenotypic data (Furbank and Tester, 2011). High throughput phenotyping technologies, which enable rapid and efficient measurement of physical traits in organisms, are increasingly being developed to bridge this gap (Herr et al., 2023). These include, but are not limited to, phone apps, automated lab equipment and greenhouses, RGB and hyperspectral imaging technologies, light detection and ranging (lidar) scanners, and ground penetrating radar (De Bei et al., 2016; Jimenez-Berni et al., 2018; Siebers et al., 2018; Bai et al., 2019; Ferguson et al., 2021; Jin et al., 2021; Manavalan et al., 2021; Montes et al., 2022; Schuhl et al., 2022). These technologies have advanced beyond mere data collection by facilitating a convergence of expertise from multiple disciplines, which enables the affordable and rapid transformation of raw data into biologically meaningful traits.

Despite these advancements in high throughput phenotyping, a gap in robust statistical design persists, hampering the adoption of newer, better, cheaper or readily available technologies. Existing reviews of technological improvement often compare methods and associated phenotypic values that neither indicate methodological quality nor permit cross-study comparisons (Grzybowski et al., 2021; Fu et al., 2022). These limitations can be ameliorated through experimental designs and statistical tests that compare both bias and variances, which has been the standard in statistics for the last six decades (Bruning and Kintz, 1968; Bethea et al., 1975; Blank, 1980; Dixon and Massey, 1983; Ott and Longnecker, 2015; Taffé et al., 2020; Yen et al., 2020), leading to unbiased and objective assessment of new methods.

The prevailing issue with existing approaches to assessing method quality lies in their failure to account for variance. Although Pearson’s correlation coefficient (*r*) and Limits of Agreement (LOA) are commonly used, both are flawed for the purpose of method comparison (De Bei et al., 2016; Madec et al., 2017; Siebers et al., 2018; Zhang et al., 2020; Taffé, 2021). Specifically, *r*, despite its intuitive appeal, is a measure of strength of a linear relationship between two variables but does not quantify the variability within each method. Stated differently, it assesses whether two techniques are measuring the same thing, but does not determine the precision of either method. Hence, a large *r* indicates that two methods measure the same thing, but does not indicate whether either method measures that thing well (see **Supplementary Section S1** for more discussion). Similarly, the LOA method, despite being one of the most cited papers for method comparison (Van Noorden et al., 2014), also fails to test which method is more variable and offers a potentially misleading binary judgment based on predetermined thresholds. Consequently, one might improperly reject a more precise method or accept a less accurate one. This is not an issue of statistical power, and increasing the sample size of the experiment does not resolve this issue.

Comparative statistical analyses between a novel method and the established “gold-standard” should rigorously evaluate both the accuracy and precision of each method over a range of values. Accuracy refers to the degree to which the “true value” (µ) is approximated by measurement (see **Supplementary Section S2** for symbol meanings). When µ is known, it is quantified as bias ( $\hat{b}$ ), and a low bias indicates high accuracy. When µ is not known, bias between the two methods ( ${\hat{b}}^{AB}$ ) is calculated instead. A low ${\hat{b}}^{AB}$ suggests that both methods yield, on average, comparable results. In addition, precision reflects the variability in repeated measurements of an identical subject, such as a specific plot, plant or leaf. This is quantified as variance, which is the sum of squared differences between individual measurements and a method’s mean estimate. A low variance signifies high precision. While bias can be estimated in typical experimental designs, estimating variance requires multiple measurements of the same subject, a feature often neglected in current experimental setups (For a more detailed explanation of bias and variance see **Supplementary Section S2**).

Statistical tests comparing bias and variances of two methods are straightforward to conduct. A significant difference in bias between two methods is indicated if ${\hat{b}}^{AB}$ is significantly different from zero as determined by a two-tailed, two-sample t-test. Variances are considered different if the ratio of the estimated variances ( ${\hat{\sigma }}_{A}^{2}/{\hat{\sigma }}_{B}^{2})$ is significantly different from one as indicated by a two-tailed F test. These statistical tests are supported by most statistical software packages. They can also adapt to varying levels of bias and variance across a range of µ values (Giavarina, 2015).

A common goal in phenotyping is to develop a way to predict a hard-to-measure “ground-truth” trait from measurements using a new, easier method. For example, researchers are able to predict photosynthetic capacity from hyperspectral scans of leaves instead of using gas exchange instruments (Yendrek et al., 2017; Meacham-Hensold et al., 2020; Montes et al., 2022). Such studies use various types of statistical models, with the easily-measured trait, or traits (for example, hyperspectral scans), as independent variables, and the ground-truth trait (for example, gas exchange results) as the dependent variable. The ground-truth trait must be measured to develop the model, but once developed, only the easily-measured trait needs to be collected.

Developing an appropriate ground-truthing model involves calculating statistics that quantify the deviations of the model predictions from measurements of the ground-truth trait. Commonly used statistics for this purpose are the root mean square error (RMSE) and mean absolute error of model predictions, and Willmott’s index of agreement. These model comparison statistics are necessary to identify appropriate models, and in this context, *r* is also a useful statistic, but in themselves these statistics do not provide sufficient information about the relative quality of the two methods. The models are parameterized in a way that minimizes the mean differences between the observations and the models predictions, which intuitively serves to minimize bias, addressing one aspect of method quality. However, the RMSE of the model error conflates information of the variances of each method, such that it cannot be used to determine which method is more precise. For example, a low model RMSE indicates that both methods are reasonably precise, but that does not imply that the new method is more precise than the old one. Conversely, a large model RMSE could indicate that either or both methods are imprecise. If the poor fit is due solely to an imprecise old, hard-to-measure method, one may mistakenly conclude that the new modeled method is inferior. Not all method development requires such model building - the methods presented below do not - but for those that do, these statistics are necessary but incomplete, because they do not quantify precision.

The primary objectives of this study are (1) to outline a rigorous statistical framework for method comparison focused on testing bias and variance rather than using *r* or LOA and (2) to empirically demonstrate the utility of this framework through case studies involving high-throughput phenotyping methods. These objectives will be tested by comparing “gold-standard” methods of canopy height and leaf area index (LAI) with high throughput phenotyping tools and algorithms of our own design. We conducted repeated measurements of canopy height, LAI-2200 measurements of leaf area, and lidar scans in sorghum (*Sorghum bicolor*) at a variety of growth stages. To further demonstrate the general usefulness of testing bias and variance, we also reanalyzed the data set used in the original manuscript describing the LOA technique (Bland and Altman, 1986) and show that the approach incorrectly rejected a new method. By adopting this refined statistical approach, we aim to overcome current limitations hindering method adoption, thereby significantly accelerating the pace of scientific discovery. This will make cross-study comparisons more reliable and enhance the overall efficiency and effectiveness of high-throughput phenotyping.

## 2 Methods

### 2.1 Lidar data collection

The data collection system consisted of a lidar scanner (UST-10LX, Hokuyo Automatic CO., LTD., Osaka, Japan) and router, powered by a battery (Sherpa 100, GoalZero, Bluffdale UT), mounted on a cart. A laptop connected to the router and data were collected using open source software (UrgBenri Standard V1.8.1, https://sourceforge.net/projects/urgbenri/). The lidar has a 90 degree blind spot that was mounted facing downward. It emits pulses of far red (905 nm) light at 40 Hz in a 270 degree sector with an angular resolution of 0.25 degrees. The maximum range of the lidar is 30 m and the precision is ± 40 mm.

#### 2.1.1 Experimental design

In 2018, 2019 and 2020 staggered-planting experiments were conducted at the University of Illinois Energy Farm (Urbana, IL 40.065707°, -88.208683°). **Supplementary Table 1** shows the number of energy sorghum (*Sorghum bicolor*) varieties planted each year and when they were planted. A series of plots were planted on successive dates so that at the end of the growing season plots would have a chronological series of heights and LAIs. Generally, after the first planting, each successive planting was done a month later. Plots were sown using a precision planter at 25 seeds per meter and fertilized with urea at a rate of 180 lbs/acre. Every year, plots consisted of four, 10 foot long rows with 30 inch row spacing. A three foot alley was left between each variety (**Supplementary Figure 1**).

#### 2.1.2 Data collection

In 2018, 2019 and 2020 repeated measurements of height and LAI were taken in a single day (**Supplementary Table 1**). Given a standard set of instructions, five individuals measured height and LAI in every plot. Plant height was considered the average height at the top of the plants. Individuals were told to imagine, “a weightless plane of styrofoam resting on the top of the plants and to measure the height of that plane.” Plant height was measured in meters using a tape measure in 2018 and 2019. In 2020 a digital ruler was used for plots greater than 1.5 m in height (Nedo mEsstronic Easy, Nedo Germany). LAI is a dimensionless measurement that quantifies m^2^ of leaf area per 1 m^2^ of ground area. It was measured using a canopy analyzer (LAI-2200, LI-COR Biosciences Lincoln, NE). Using a 45 degree view-cap, two sets of above-leaves and below-leaves measurements were made for each plot following guidelines provided for row crops (LiCOR 2200c Manual, section 6-4). Every year, five people shared two LAI-2200 instruments.

The lidar cart was run through every plot five times in 2018, 2019 and 2020. Data were collected at approximately 1 m/s. The data were processed for height and LAI using the algorithms described below.

### 2.2 Algorithms

Lidar data were processed before running the height and LAI estimation algorithms. The lidar gives coordinates in a polar coordinate system (θ: the angle within the plane that the lidar scans; *r*: the straight-line distance from the center of lidar). Assuming that the lidar was level and a fixed height above the ground, lidar returns were transformed to a Cartesian coordinate system (*x*: the horizontal distance from the lidar; *y*: the vertical distance above the ground). Next all *x* points greater than 30 inches left and right of the lidar were removed. This ensured that only the middle two rows of energy sorghum were being analyzed. Additionally the first and last 15 percent of scans were removed to avoid analyzing data associated with the edge of the plots. To remove remaining outliers, Grubbs’s test (Grubbs, 1950) was used (*α* = 0.01, smirnov_grubbs function from the outliers module; Python 3.6.5) on the distribution of all vertical distances (*y*, in the Cartesian coordinate system) for a plot. Crop height and LAI were then calculated on these processed data. Height was estimated as the 99th percentile of *y* values in a plot.

Lidar LAI was estimated on the processed data using the gap fraction method (Miller, 1967). To calculate gap fraction, angles were grouped into intervals with the following boundaries: 0, 15, 30, 45, 60 and 90 degrees, where 0 degrees is upward. The ratio of the number of angles without a lidar return to the total number of angles in that interval was used as the gap fraction. Occasionally, all angles in an interval were intercepted by a plant, and thus the gap fraction was 0. This results in the log of 0, which is undefined. In such cases, the gap fraction was replaced with 1/4 of the gap fraction in the next skyward interval, which is an arbitrary decision that approximates the observed relationship of gap fraction and zenith angle.

### 2.3 Data from Bland and Altman (Bland and Altman, 1986)

Data from the table in Bland and Altman (Bland and Altman, 1986) were reanalyzed in order to compare variances. To briefly describe their measurements in that study, peak expiratory flow rate (PEFR) was measured with a Wright peak flow meter and a “mini” Wright peak flow meter. Two measurements using each meter were made on each of 17 subjects.

### 2.4 Bias and variance estimates

The following model was used for each method:


$$y_{ij}^{M}=\mu_{j}+b^{M}+e_{ij}^{M}$$

where *j* is the plot ID (a.k.a subject) from 1 … *k*, which varied by year, and *i* is the observation ID from 1 … *n^M^*, which varied by method and year (**Supplementary Table 1**). Residuals were assumed to be independent and to come from a distribution with mean 0 and either constant variance $\sigma_{M}^{2}$ for LAI, or for height, a separate variance for each plot $\sigma_{M,j}^{2}$ . Nonhomogeneity of variances was determined by visual inspection, presented below. Variances of each method were estimated as the within-subject variance.

To choose predetermined limits of agreement thresholds for LAI and height, we decided that the disagreement of the bias should be no greater than 5 percent of the greatest values (LAI of about 6; height of about 3.5 m). Thus, the thresholds for limits of agreement of -0.3 to 0.3 were chosen for LAI and -0.18 to 0.18 m for height. For peak expiratory flow rate, the threshold in the original paper is not stated, but the authors rejected LOA of -80 to 76 l/min (Bland and Altman, 1986). Thus, we chose a threshold of 80 l/min, the larger limit in absolute value.

### 2.5 Statistical tests

Bias was assessed using a two-tailed t-test of whether bias = 0 with α = 0.05. Variances were compared using a two-tailed F-test of whether ${\hat{\sigma }}_{A}^{2}/{\hat{\sigma }}_{B}^{2}>1$ , with α = 0.05. For height and LAI estimates, variances varied with the mean and therefore variances were estimated and compared for each plot. For PEFR, variances were compared for each subject, but since variances did not scale with the mean, variances of all subjects for a single method were pooled into a single variance for each method. Those pooled variances were compared using the same F-test as above.

### 2.6 Data availability

Code for the open-source statistical software R is included in the **Supplementary Material**. The code can be used to reproduce the results and figures. It can also be used as a template for analyzing repeated measurements. All of the data used in the analysis are included as .csv files.

## 3 Results

### 3.1 Lidar estimations of plant height were unbiased and less variable than the manual method

**Figure 1:**
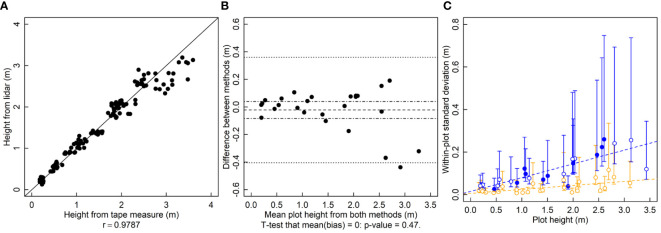
Plant height (height, m) measured by a lidar and a tape measure. **(A)** A correlation plot of height estimates of the two methods. **(B)** Bias plot with mean bias (dashed line) standard error of the bias (dot-dashed line) and limits of agreement (dotted line). **(C)** Within-plot variances for each method against the mean height, where blue symbols are for the tape measure and orange symbols are for lidar-estimated height. Filled symbols indicate a significantly larger variance for that method for that subject, and open symbols indicate no significant difference or significantly smaller (two-tailed F-test that the variances are equal; α = 0.05). Dashed lines are ordinary least squares regression fits to the data, but are for visualization only since the chi-squared distribution of the variances is highly non-normally distributed, violating regression assumptions.

To calculate *r* for the height results, tape measure heights are randomly paired with a lidar measurement from the same plot. In a given plot there were five tape measure heights and five lidar heights. The *r* between tape measure height and lidar height is 0.98 and the relationship falls near the 1-to-1 line (**Figure 1A**). For estimating bias, differences between the means of the five repeated measurements from each method are calculated. There are no serious indications of bias between the lidar and tape measure heights (p = 0.47, **Figure 1B**). The variance of the within-plot repeated lidar estimates of height is lower than that of tape-measure estimates for most measurements (**Figure 1C**). The variance of both lidar and tape-measure manual methods of height increases in taller crop stands (**Figure 1C**).

### 3.2 Lidar estimations of LAI were biased and more variable than the canopy analyzer

**Figure 2:**
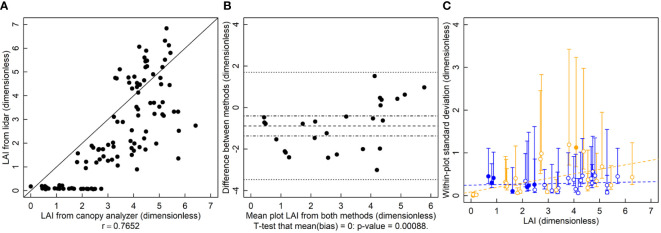
Leaf area index (LAI, dimensionless) measured by a lidar and canopy analyzer. **(A)** A correlation plot of LAI estimates of the two methods. **(B)** Bias plot with mean bias (dashed line) standard error of the bias (dot-dashed line) and limits of agreement (dotted line). **(C)** Within-plot variances for each method against the mean LAI, where blue symbols are for the LAI-2200 meter and orange symbols are for lidar estimated LAI. Filled symbols indicate a significantly larger variance for that method for that subject, and open symbols indicate no significant difference or significantly smaller (two-tailed F-test that the variances are equal; α = 0.05). Dashed lines are ordinary least squares regression fits to the data, but are for visualization only since the chi-squared distribution of the variances is highly non-normally distributed, violating regression assumptions.

The *r* is 0.77 and the relationship between the LAI-2200 and the lidar deviates from the 1-to-1 line (**Figure 2A**). Lidar LAI estimates were significantly biased and lower than those of the canopy analyzer (**Figure 2B**, p=0.0009). Estimates of variance showed no indication of serious deviation from homogeneity. The variance of lidar-based LAI estimates is greater than those of the canopy analyzer for several of the plots with LAI values below 3 (**Figure 2C**).

### 3.3 Mini-Wright estimations of peak expiratory flow rate (PEFR) were unbiased and equally variable as the Wright meter. The LOA were outside the acceptable threshold

**Figure 3:**
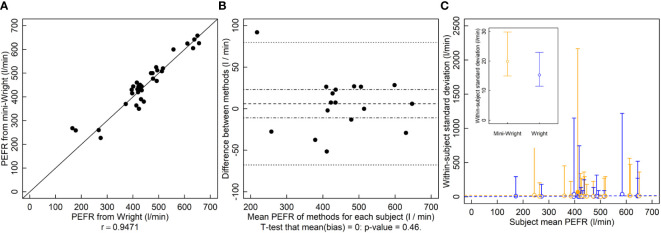
Peak expiratory flow rate (PEFR, l/min) measured by the Wright and mini-Wright peak flow meters. **(A)** A correlation plot of PFER estimates from each method. Source: Based on data from Bland and Altman (1986). **(B)** Bias plot with mean bias (dashed line) standard error of the bias (dot-dashed line) and limits of agreement (dotted line). **(C)** Within-patient variances against subject mean where blue symbols are data for the Wright meter and orange symbols are for the mini-Wright meter. Filled symbols indicate a significantly larger variance for that method for that subject, and open symbols indicate no significant difference or significantly smaller (two-tailed F-test that the variances are equal; α = 0.05). One filled blue symbol is occluded by other symbols near PFER=400. (C, inset) Pooled variances with their 95% confidence limits. The pooled variances were not significantly different by a two-tailed F-test of equal variances using α = 0.05. Dashed lines are ordinary least squares regression fits to the data, but are for visualization only since the chi-squared distribution of the variances is highly non-normally distributed, violating regression assumptions.

The *r* between the Wright and Mini-Wright instruments was 0.95 and the relationship falls near the 1-to-1 line (**Figure 3A**). The LOA were -68 l/min to 80 l/min (**Figure 3B**) which exceeded the predetermined threshold. The analysis in the original Bland and Altman manuscript reports LOA of -80 l/min to 75 l/min. The original analysis uses only one set of samples, whereas the analysis here uses both sets. It also calculates the difference “Wright minus mini-Wright”, whereas here “mini-Wright minus Wright” is used in order to match “new method - old method” used for the height and LAI analyses, hence the change in sign and slight difference in LOA here compared to the original paper. There was no indication of bias (**Figure 3B**). Paired comparisons of variances for each subject showed that the mini-Wright had larger variance for one subject and smaller variance for another, with no differences in the rest of the 17 subjects (**Figure 3C**). The conflicting results, and few significant values relative to the number of subjects suggested type I errors due to low power. Variances did not appear to depend on the mean, indicating that they could be pooled to increase power. Thus, variances across subjects were pooled. Using the pooled variances, there was no indication that variances of methods were different (**Figure 3C** inset, p = 0.28).

### 3.4 Summary of interpretations

Interpretations of results for Pearson’s correlations coefficient would lead any researcher to accept the new height estimation method, but likely researchers would find it difficult to make firm conclusions about lidar-based LAI estimate (**Table 1**). Furthermore, for neither height nor LAI does the correlation coefficient give information about the relative quality of the two methods.

**Table 1:** A summary of interpretations for each method comparison using each of the statistical approaches.

Approach	Lidar Height	Lidar LAI	Wright Flow Meter
Correlation coefficient	Accept	Unclear	Accept
Limits of agreement	Reject	Reject	Reject
Bias and variance	Lidar is unbiased and substantially more precise	Lidar is biased and possibly less precise at meaningful LAIs	The mini flow meter is unbiased and equally as precise

Comparing bias and precision, lidar estimations of height are unbiased and more precise. Lidar-based LAI estimates are biased and except at the lowest height, the instruments do not differ in precision (**Table 1**).

## 4 Discussion

The field of phenomics has no consistent standard for statistical method comparison. The use of *r* is appealingly simple but offers no insight into method quality. Although it has been discussed for use in remote sensing, the popular alternative, LOA, should not be used because it fails to identify which method is more variable (Coops et al., 2021). Instead studies should be designed to perform repeated measurements of subjects to test bias and variance across a range of values. The interpretations of *r* and LOA for height, LAI and the original data used to describe LOA demonstrates the improvement of using formal statistical tests of bias and variance; the interpretation of *r* for the LAI methods is unclear and LOA reaches the wrong conclusion for the height and flow meter methods. The improved ability to objectively assess method quality will accelerate and inform method adoption in plant phenomics.

The largely arbitrary nature of using *r* to determine whether a new method is a suitable substitute for an old one (**Table 1**) is demonstrated by the LAI analysis. In this case, the *r* for these correlations was about 0.77. Are these values large enough to be considered acceptable? Some may consider these large enough that lidar could acceptably replace the canopy analyzer. Others may disagree. There is not a clear choice for an acceptably large *r* and thus no statistical test to determine whether observed results exceed that threshold. For lidar estimates of height, the *r* value was 0.98. These would be considered an excellent correlation. These conclusions are unfulfilling though. Why does the correlation appear to degrade at higher heights? Is it wind? Does the lidar fail to see the top of the canopy? What one wants to know is which method is better and in what way. One may conclude that because *r* is very close to 1 that these questions are irrelevant and the methods are equivalent, when in fact the lidar height estimation method is considerably better than the tape measure method.

LOA is used pervasively in the place of *r.* However it should be avoided. In the examples shown here, LOA rejects a superior method (lidar based height), does not allow the conditional use of another (lidar based LAI) and also rejects an equivalent, possibly cheaper method (mini-Wright meter). The central issue with LOA is that the variance of the bias contains the sum of the variance of both methods (Bland and Altman, 1999). Therefore, using only limits of agreement, it is difficult to accept a new method when the established, ground-truth method is highly variable or if the variability of the ground-truth method is already close to the threshold of agreement. The variance of the ground-truth method alone can push the methods outside of the limits of agreement. Here the lidar-based height estimation method was rejected because the limits of agreement (-0.4 to 0.36 m) were outside of the predetermined threshold of 0.18 m (**Figure 1B**), which is large enough to be meaningful for many researchers (Sabadin et al., 2012). However, not adopting lidar-based estimates of height would clearly be a mistake, as the new method has much smaller variance and no bias. The limits of agreement approach rightfully identifies that the two methods do not agree, but it misses the fact that the disagreement is because the old method is inferior to the new one. The logical flaw in using LOA is in assuming that the so-called ground-truth method is in fact the truth, but “Which method is better?” is the very question these experiments are meant to answer. A statistical method that assumes that the existing method is the “right” one makes it logically impossible to properly address the question. In contrast, the use of an F-test doesn’t make such assumptions and makes it clear which method is more variable (**Figure 1C**). For the height estimation methods, using limits of agreement results in rejecting a clearly superior method.

This shortcoming in the limits of agreement approach was identified previously and measuring the coefficient of repeatability was suggested (Bland and Altman, 1986). Like variance, this statistic is a measure of precision and requires repeated measurements of a subject. As an example, Bland and Altman (Bland and Altman, 1986) compare two PEFR meters: one full sized (Wright) and one miniaturized version (mini-Wright). Based on their calculated limits of agreement, the instruments often differed by more than 80 l/min, which they considered an unacceptable difference (**Figure 3B**). They then calculated the coefficient of repeatability of each instrument, finding 43 l/min for the Wright and 56 l/min for the mini-Wright. Since the coefficient for the mini-Wright was larger, they then concluded that the disagreement stems from the variability of the mini-Wright meter. However, no statistical test was performed to compare the coefficients. Like other estimates, variance estimates have a degree of randomness, and that one is larger than the other does not necessarily indicate a true difference. Thus, tests must be performed to provide a degree of confidence in their difference.

Here, reanalyzing the data and comparing variances of paired measurements of each subject, we found no apparent difference in variances of the Wright and mini-Wright meters (**Figure 3C**). Confidence limits overlap substantially. The very large confidence limits are because measurements were replicated only twice for each subject, which results in poor statistical power. However, given that there is no apparent relationship between the variance and the mean for either method, the variances of different subjects can be pooled, giving smaller confidence limits and better statistical power to determine differences between the methods. When pooling the data, there is still no indication that the mini-Wright meter is more variable than the Wright meter (**Figure 3C**, inset; p=0.28, two-tailed F-test of ${\hat{\sigma }}_{mini}^{2}/{\hat{\sigma }}_{Wright}^{2}\neq 1$ ).

In this case, the limits of agreement are large because neither the Wright nor mini-Wright instruments have good repeatability (**Figure 3C**, inset), not, as was originally concluded, because the mini-Wright meter is an unacceptable replacement for the Wright meter. The only way to demonstrate this is by comparing variances using statistical tests. The limits of the agreement test alone cannot determine this, and comparing measures of variability, such as the coefficients of repeatability, without using a test can lead to incorrect conclusions, as happened with these PEFR meters. The conclusion here is that the mini-Wright flow meter is unbiased and has equal variance compared to the Wright meter, so its quality is equivalent to that of the Wright meter.

There is a question of how often this flaw of limits of agreement could result in incorrect conclusions. The limits of agreement approach makes it possible to (1) reject a new more precise method, (2) reject a new, equivalent method or, (3) accept a new, less precise method. Most method assessment studies do not collect the data required to determine variance, so a comprehensive review of literature is not possible. However, the authors hardly had to search to find examples. It occurs with our height data set, where a newer more precise method is rejected. Outside of our own data, the first study we considered, the original paper describing limits of agreement, has the same limitation. The mini-Wright meter is rejected although it is an equivalent method; it is unbiased and of equal variance compared to the Wright flow meter. Moreover, if the variance of the old measurement method is very low, it is also possible to accept a new, less precise method, a situation that is potentially worse if the users are unaware. Surely there are cases where decreased precision is acceptable if the method is considerably cheaper or faster, but the limits of agreement approach cannot provide this information. A less precise method that passes the limits of agreement test would be incorrectly deemed equivalent to the existing method, and if the new method is more expensive and slower, using it would clearly be a mistake. Thinking of method comparison studies in general, a common reason to replace an established method is that it is considered inadequately precise. That is the situation in which large variance of the established method would make it difficult to accept a new method when using limits of agreement, suggesting that the problem is common. Since approximately 50,000 manuscripts cite this approach, it seems very likely that often there are new, better, methods that are incorrectly rejected.

Of course accuracy and precision are only two factors to consider among many when assessing which method is appropriate. Speed, ease of use, cost, availability and maintenance are several aspects that could be important to a researcher. Quantifying variance provides one piece of information among many for researchers to make informed decisions about methods that cannot be provided with *r* or limits of agreement. For example, lidar is an order of magnitude faster at measuring LAI than the canopy analyzer. So, even though it is more variable and biased, one could use lidar-based LAI estimates to identify overall best performers in a large field trial, and subsequently use the more precise canopy analyzer to identify the single best performer from that smaller set.

Quantifying variance also provides the ability to compare results across studies. For example, a later study of a new height-estimation method could compare variance of their method to the variances reported here. Although bias could be compared across studies if a suitable reference is identified, a reference is not sensible for all methods, as is the case here. Regardless, as described above, bias is often not a concern, whereas precision is always a consideration for method quality. Some studies compare *r*, *r* ^2^ or *R* ^2^ across experiments, but unlike variance and bias, each of these statistics depends on the experimental design, and comparing them across studies confounds differences in method quality with the experimental design, even when studies compare the same methods (Goodwin and Leech, 2006). Thus, if the studies vary in treatment size, number of replicates, or nearly any aspect of experimental design, summarizing these statistics across studies does not provide meaningful information, yet these comparisons are made (Siebers et al., 2018; Zhang et al., 2020; Grzybowski et al., 2021).

Regarding designs to compare methods, it is helpful to have a wide range of values, so that bias and variance can be assessed across all expected values. It is critical to note that variance estimates themselves are highly variable, and a small number of measurements will give poor confidence intervals, as seen in **Figure 3C**. An appropriate number depends on how variable the measurements are, which is of course not known beforehand. Thus, although the minimum required to perform the computation is only two replicates, more are almost certainly required to be practically useful. Based on the authors’ experience, 4 degrees of freedom is a minimum starting point. If estimates cannot be pooled, that requires 5 replicates per subject. It is advisable to do initial work to estimate roughly how variable the methods are and choose the number of replicates based on that.

Studies that develop ground-truthing models, such as predicting photosynthetic capacity from hyperspectral scans, should still conduct method comparison experiments. After the models are developed, the existing and new methods should each be used to collect multiple measurements of the same subjects and compared as described above. For these analyses, the data are not used to develop the models, and instead the two methods are compared as if they were independent. In the case of hyperspectral imaging, the variability of the prediction model will stem from variability of the cameras and the collection technique. In this case, it’s important to make the collection technique clear to facilitate cross-study comparison. Scanning 1 m^2^ of a plot will likely result in a different variance from scanning 10 m^2^. Despite the prediction models being chosen so that they reduce bias between the model output and ground-truth data set, potential bias could still exist, particularly if the ground-truth data set is small, in which case it could substantially deviate from the mean response. A model trained using such data would then be biased. Follow up studies using repeated measurements provide a statistical test to demonstrate a true lack of bias.

The variance tests presented in this study are the textbook approaches for method comparison, although they are not commonly used in plant phenotyping. Moreover, testing variance through repeated measurements is valuable for experiments in every discipline. A test of variance is more meaningful for interpreting the quality of a method than *r* or the limits of agreement alone. It will allow for the adoption of new methods in situations where the old method is highly variable. Testing bias and variance will help alleviate the method adoption bottleneck that is slowing the use and objective understanding of new phenomics techniques.

## 5 Conclusions

Few phenotyping method comparison studies use well established statistical analyses to test bias and variances. Such inconsistent statistical practices have likely slowed the rate of method adoption in high-throughput phenotyping and misled comparisons. Comparing bias and variance using repeated measurements is the long-standing, standard statistical approach for method comparison. It could be readily applied in phenotyping. Although *r* is useful for model development, it has previously been shown that *r* is inappropriate for method comparison. Similarly, although the use limits of agreements approach is popularly used for method comparisons, here we show that it can reject a more precise method, reject an equivalent method or even accept a less precise method, not as a matter of lack of careful interpretation, but due to an inherent deficiency in the approach. Two examples are given here - lidar-based height and the mini-Wright peak flow meter - where the limits of agreement approach leads to the incorrect conclusion and prevents the adoption of an equivalent or better method. We demonstrate examples of the standard approach by quantifying variance in order to compare methods, showing that the approach here is superior to both *r* and limits of agreement. We also provide the code, in the free programming language R, to analyze bias and variance and reproduce graphs like the ones shown in the paper.

## Data availability statement

The original contributions presented in the study are included in the article/**Supplementary Material**. Further inquiries can be directed to the corresponding author.

## Author contributions

JM: Writing – original draft, Writing – review & editing, Conceptualization, Data curation, Formal analysis, Investigation, Methodology, Project administration, Resources, Software, Supervision, Validation, Visualization. MS: Writing – original draft, Writing – review & editing, Conceptualization, Formal analysis, Investigation, Methodology, Project administration, Software, Supervision, Validation, Visualization. PF: Writing – review & editing, Data curation, Formal analysis, Methodology, Software, Validation. SL: Writing – review & editing, Funding acquisition, Project administration, Resources, Supervision. CB: Writing – review & editing, Project administration, Resources, Supervision, Validation.
