# Supplementary material for: To have value, comparisons of high-throughput phenotyping methods need statistical tests of bias and variance
Source: Front Plant Sci. 2024 Jan 19;14:1325221. doi: 10.3389/fpls.2023.1325221 (PMC10835710; doi:10.3389/fpls.2023.1325221)
Supplement: Supplementary file 5 [file DataSheet_5.docx]

####################

# File description #

####################

# This file contains the code necessary to reproduce the analyses and figures in the manuscript.

# R version 4.2.1 was used in the manuscript, but other versions will likely work.

# The file starts with function definitions for analyses shared by the three comparisons.

# The analyses are performed after the function definitions.

########################

# Function definitions #

########################

subject_stats = function(data, value_name, subject_names, alpha=0.05) {

# For each subject, calculate the mean, sample size, degrees of freedom, variance and confidence limits of the variance.

# Returns a data frame of these stats with a row for each subject.

result = as.data.frame(as.list(aggregate(data[value_name], data[subject_names], function(x) c(mean=mean(x), var=var(x), n=length(x)))))

result = setNames(result, c(subject_names, 'mean', 'var', 'n'))

result = within(result, {

df = n - 1

var.lower.cl = var * df / qchisq(1 - alpha / 2, df)

var.upper.cl = var * df / qchisq(alpha / 2, df)

})

return(result)

}

variance_comparisons = function(old_data, new_data, value_name, subject_names) {

# For each subject, compare varances of the old and new methods.

# Returns a data frame of these comparisons with a row for each subject.

subjects = unique(rbind(old_data[subject_names], new_data[subject_names]))

# There is not a convenient function to subset a data frame based on a matching row.

# The `match_rows` function finds rows in `data_frame` that match a single row given by `row_to_match`

# and returns a vector of logical values indicating whether a row in data_frame matches.

match_rows = function(data_frame, row_to_match) {

n = nrow(data_frame)

result = logical(n)

for (i in seq_len(n)) {

result[i] = all(data_frame[i, names(row_to_match)] == row_to_match)

}

result

}

n_subjects = nrow(subjects)

result = vector('list', n_subjects)

for (i in seq_len(n_subjects)) {

subject = subjects[i, , drop=FALSE]

one_new_subject = new_data[match_rows(new_data, subject), ]

one_old_subject = old_data[match_rows(old_data, subject), ]

result[[i]] = cbind(subject, as.data.frame(as.list(unlist(var.test(one_new_subject[[value_name]], one_old_subject[[value_name]])[c('statistic', 'conf.int', 'parameter', 'p.value')]))))

}

result = do.call(rbind, result)

return(result)

}

bias_analysis = function(old_data, new_data, value_name, subject_names) {

# For each subject, calculate the overall mean of the two methods and bias between the methods.

# Returns a data frame of these stats with a row for each subject.

wide_data = merge(old_data[c(subject_names, value_name)], new_data[c(subject_names, value_name)], by=subject_names)

col_names = paste(value_name, c('x', 'y'), sep='.')

wide_data$overall_mean = apply(wide_data[col_names], 1, mean)

wide_data$bias = wide_data[[col_names[2]]] - wide_data[[col_names[1]]]

return(wide_data)

}

loa = function(old_data, new_data, value_name, subject_names) {

# Calculate limits of agreement for repeated measurements with unequal samples.

# Returns a vector with the limits of agreement.

# This is from Bland and Altman (1999) Measuring agreement in method comparison studies.

# Statistical Methods in Medical Research. 8: 135-160.

old_subject_stats = subject_stats(old_data, value_name, subject_names)

new_subject_stats = subject_stats(new_data, value_name, subject_names)

var_old = with(old_subject_stats, sum(var * df) / sum(df))

var_new = with(new_subject_stats, sum(var * df) / sum(df))

biases = bias_analysis(old_subject_stats, new_subject_stats, 'mean', subject_names)

mean_bias = mean(biases$bias)

var_bias = var(biases$bias)

n = nrow(old_subject_stats)

coef_old = (1 / n) * sum(1 / old_subject_stats$n)

coef_new = (1 / n) * sum(1 / new_subject_stats$n)

var_mean_bias = var_bias + (1 - coef_old) * var_old + (1 - coef_new) * var_new # Equation 15.3 of Bland and Altman 1999.

loa = mean_bias + 1.96 * c(-sqrt(var_mean_bias), sqrt(var_mean_bias))

return(loa)

}

variance_plot = function(old_mean, old_var, old_lower, old_upper, new_mean, new_var, new_lower, new_upper, F, p) {

# Choose open or closed circles depending on statistical significance

old_is_larger = (p <= 0.05 & F < 1) + 1

new_is_larger = (p <= 0.05 & F > 1) + 1

orange_fills = c('white', 'orange')

blue_fills = c('white', 'blue')

# The arrows are plotted separately from the points.

# The arrows are drawn first so that they points will cover them

# where necessary. The following command creates the plotting area

# but doesn't drawn any data so that the arrows can be drawn first.

# Axes are also not draw so that they can be customized.

par(tck=0.02, mar=c(3.25, 2.25, 0.25, 0.25))

plot(sqrt(new_var) ~ new_mean,

main=NULL,

col='orange',

yaxt='n',

xaxt='n',

ylim=range(pretty(sqrt(c(old_lower, old_upper, new_lower, new_upper)), high.u.bias=2)),

xlim=range(pretty(c(0, old_mean, new_mean)), high.u.bias=2),

ylab='',

xlab='',

bg=orange_fills[new_is_larger],

type='n',

pch=21)

x_labels = axTicks(1)

axis(1, at=x_labels, label=rep("", length(x_labels)))

axis(1, at=x_labels, tck=0, lwd=0, line=-0.7)

y_labels = axTicks(2)

axis(2, at=y_labels, label=rep("", length(y_labels)))

axis(2, at=y_labels, tck=0, lwd=0, line=-0.7)

arrows(new_mean, sqrt(new_upper),

new_mean, sqrt(new_lower),

length=0.03, angle=90, code=3,

col='orange')

arrows(old_mean, sqrt(old_upper),

old_mean, sqrt(old_lower),

length=0.03, angle=90, code=3,

col='blue')

points(sqrt(old_var) ~ old_mean,

col='blue',

bg=blue_fills[old_is_larger],

pch=21)

points(sqrt(new_var) ~ new_mean,

col='orange',

bg=orange_fills[new_is_larger],

pch=21)

# Plot regression lines with the caveat that these are for a

# general understanding. Assumptions of normality are fairly strongly

# violated since variances are chi-squared distributed.

abline(coef(lm(sqrt(new_var) ~ new_mean)), col='orange', lty=2)

abline(coef(lm(sqrt(old_var) ~ old_mean)), col='blue', lty=2)

}

bias_plot = function(bias, means, loa, alpha=0.05, ...) {

# Confidence limits for bias. Bias is t distributed with n - 1 degrees of freedom.

bias_mean = mean(bias)

cl = qt(alpha/2, df=length(bias) - 1, lower=FALSE)

bias_sd = sd(bias)

bias_se = sd(bias) / sqrt(length(bias))

upper_sd = bias_mean + bias_sd * cl

lower_sd = bias_mean - bias_sd * cl

upper_se = bias_mean + bias_se * cl

lower_se = bias_mean - bias_se * cl

pvalue = pt(abs(bias_mean) / bias_se, length(bias) - 1, lower=FALSE) * 2

xlimits = range(pretty(means))

ylimits = range(pretty(c(bias, loa)))

max_abs = max(abs(ylimits))

ylimits = c(-max_abs, max_abs) # Keep the axes symmetrical to ease interpretation.

par(tck=0.02, mar=c(3.75, 2.25, 0.25, 0.25))

plot(bias ~ means,

pch=19,

ylim=ylimits,

xlim=xlimits,

ylab='',

xlab='',

xaxt='n',

yaxt='n',

...)

abline(h=bias_mean, lty=2)

abline(h=c(upper_se, lower_se), lty=4)

abline(h=loa, lty=3)

title(sub=paste0('T-test that mean(bias) = 0: p-value = ', format(pvalue, digits=2) ,'.'), line=2.25)

x_labels = axTicks(1)

axis(1, at=x_labels, label=rep("", length(x_labels)))

axis(1, at=x_labels, tck=0, lwd=0, line=-0.7)

y_labels = axTicks(2)

axis(2, at=y_labels, label=rep("", length(y_labels)))

axis(2, at=y_labels, tck=0, lwd=0, line=-0.7)

}

r_plot = function(old_data, new_data, value_name, subject_names) {

# Creates a plot similar to those found in method comparison studies.

# Usually studies only have a single measurement per subject, and they pair those measurements.

# Here there are multiple measurements, so they are paired based on their replicate number.

# If one method has more replicates than the other, the extras are not plotted.

old_data$replicate = ave(old_data[[value_name]], old_data[subject_names], FUN = seq_along)

new_data$replicate = ave(new_data[[value_name]], new_data[subject_names], FUN = seq_along)

all_data = merge(new_data, old_data, by=c(subject_names, 'replicate'))

x_data = all_data[[paste(value_name, 'x', sep='.')]]

y_data = all_data[[paste(value_name, 'y', sep='.')]]

r = with(all_data, cor(x_data, y_data, use='complete.obs'))

par(tck=0.02, mar=c(3.75, 2.25, 0.25, 0.25))

plot(x_data ~ y_data,

all_data,

type=c('p'),

pch=19,

ylab='',

xlab='',

main=NULL,

xaxt='n',

yaxt='n',

ylim=range(pretty(c(0, x_data, y_data), na.rm=TRUE)),

xlim=range(pretty(c(0, x_data, y_data), na.rm=TRUE)))

abline(0, 1)

title(sub=parse(text=paste0('r==', round(r, 4))), line=2.25)

x_labels = axTicks(1)

axis(1, at=x_labels, label=rep("", length(x_labels)))

axis(1, at=x_labels, tck=0, lwd=0, line=-0.7)

y_labels = axTicks(2)

axis(2, at=y_labels, label=rep("", length(y_labels)))

axis(2, at=y_labels, tck=0, lwd=0, line=-0.7)

}

##################################

# Statistical analyses and plots #

##################################

###################

# Height analysis #

###################

height_person = read.csv('Data Sheet 1.CSV')

height_lidar = read.csv('Data Sheet 2.CSV')

height_person_stats = subject_stats(height_person, 'height', c('plot_id', 'year'))

height_lidar_stats = subject_stats(height_lidar, 'height', c('plot_id', 'year'))

height_comparisons = variance_comparisons(height_person, height_lidar, 'height', c('plot_id', 'year'))

height_bias = bias_analysis(height_person_stats, height_lidar_stats, 'mean', c('plot_id', 'year'))

(height_loa = loa(height_person, height_lidar, 'height', c('plot_id', 'year')))

png(filename='Height bias plot.png', height=90, width=90, res=600, units='mm', pointsize='10')

bias_plot(height_bias$bias, height_bias$overall_mean, height_loa)

title(ylab='Difference between methods (m)', line=1.25)

title(xlab='Mean plot height from both methods (m)', line=1.25)

dev.off()

png(filename='Height variance plot.png', height=90, width=90, res=600, units='mm', pointsize='10')

variance_plot(height_person_stats$mean, height_person_stats$var, height_person_stats$var.lower.cl, height_person_stats$var.upper.cl,

height_lidar_stats$mean, height_lidar_stats$var, height_lidar_stats$var.lower.cl, height_lidar_stats$var.upper.cl,

height_comparisons$statistic.F, height_comparisons$p.value)

title(ylab='Within-plot standard deviation (m)', line=1.25)

title(xlab='Plot height (m)', line=1.25)

dev.off()

png(filename='Height correlation plot.png', height=90, width=90, res=600, units='mm', pointsize='10')

r_plot(height_person, height_lidar, 'height', c('plot_id', 'year'))

title(ylab='Height from lidar (m)', line=1.25)

title(xlab='Height from tape measure (m)', line=1.25)

dev.off()

################

# LAI analysis #

################

lai_person = read.csv('Data Sheet 3.CSV')

lai_lidar = read.csv('Data Sheet 4.CSV')

lai_person_stats = subject_stats(lai_person, 'lai', c('plot_id', 'year'))

lai_lidar_stats = subject_stats(lai_lidar, 'lai', c('plot_id', 'year'))

lai_comparisons = variance_comparisons(lai_person, lai_lidar, 'lai', c('plot_id', 'year'))

lai_bias = bias_analysis(lai_person_stats, lai_lidar_stats, 'mean', c('plot_id', 'year'))

(lai_loa = loa(lai_person, lai_lidar, 'lai', c('plot_id', 'year')))

png(filename='LAI bias plot.png', height=90, width=90, res=600, units='mm', pointsize='10')

bias_plot(lai_bias$bias, lai_bias$overall_mean, lai_loa)

title(ylab='Difference between methods (dimensionless)', line=1.25)

title(xlab='Mean plot LAI from both methods (dimensionless)', line=1.25)

dev.off()

png(filename='LAI variance plot.png', height=90, width=90, res=600, units='mm', pointsize='10')

variance_plot(lai_person_stats$mean, lai_person_stats$var, lai_person_stats$var.lower.cl, lai_person_stats$var.upper.cl,

lai_lidar_stats$mean, lai_lidar_stats$var, lai_lidar_stats$var.lower.cl, lai_lidar_stats$var.upper.cl,

lai_comparisons$statistic.F, lai_comparisons$p.value)

title(ylab='Within-plot standard deviation (dimensionless)', line=1.25)

title(xlab='LAI (dimensionless)', line=1.25)

dev.off()

png(filename='LAI correlation plot.png', height=90, width=90, res=600, units='mm', pointsize='10')

r_plot(lai_person, lai_lidar, 'lai', c('plot_id', 'year'))

title(ylab='LAI from lidar (dimensionless)', line=1.25)

title(xlab='LAI from canopy analyzer (dimensionless)', line=1.25)

dev.off()

#############################################################

# Flow meters from the Bland and Altman manuscript analysis #

#############################################################

large_pefr = expand.grid(list(subject=1:17, rep=1:2))

large_pefr$value = c(494, 395, 516, 434, 476, 557, 413, 442, 650, 433, 417,

656, 267, 478, 178, 423, 427, 490, 397, 512, 401, 470,

611, 415, 431, 638, 429, 420, 633, 275, 492, 165, 372,

421)

large_pefr$method = 'large'

small_pefr = expand.grid(list(subject=1:17, rep=1:2))

small_pefr$value = c(512, 430, 520, 428, 500, 600, 364, 380, 658, 445, 432,

626, 260, 477, 259, 350, 451, 525, 415, 508, 444, 500,

625, 460, 390, 642, 432, 420, 605, 227, 467, 268, 370,

443)

small_pefr$method = 'small'

large_stats = subject_stats(large_pefr, 'value', 'subject')

small_stats = subject_stats(small_pefr, 'value', 'subject')

pefr_comparisons = variance_comparisons(large_pefr, small_pefr, 'value', 'subject')

pefr_bias = bias_analysis(large_stats, small_stats, 'mean', 'subject')

(pefr_loa = loa(large_pefr, small_pefr, 'value', 'subject'))

png(filename='PEFR bias plot.png', height=90, width=90, res=600, units='mm', pointsize='10')

bias_plot(pefr_bias$bias, pefr_bias$overall_mean, pefr_loa)

title(ylab='Difference between methods (l / min)', line=1.25)

title(xlab='Mean PEFR of methods for each subject (l / min)', line=1.25)

dev.off()

# One subject has 0 variance for one method, thus there is a warning about an

# zero-length arrow when running the code below.

png(filename='PEFR variance plot.png', height=90, width=90, res=600, units='mm', pointsize='10')

variance_plot(large_stats$mean, large_stats$var, large_stats$var.lower.cl, large_stats$var.upper.cl,

small_stats$mean, small_stats$var, small_stats$var.lower.cl, small_stats$var.upper.cl,

pefr_comparisons$statistic.F, pefr_comparisons$p.value)

title(ylab='Within-subject standard deviation (l/min)', line=1.25)

title(xlab='Subject mean PEFR (l/min)', line=1.25)

dev.off()

png(filename='PEFR correlation plot.png', height=90, width=90, res=600, units='mm', pointsize='10')

r_plot(large_pefr, small_pefr, 'value', 'subject')

title(ylab='PEFR from mini-Wright (l/min)', line=1.25)

title(xlab='PEFR from Wright (l/min)', line=1.25)

dev.off()

# One comparison indicates that the Wright has larger variance, yet another

# comparison indicates that the mini-Wright has larger variance, but it doesn't

# appear that variances depend on the mean. The contrasting results, combined

# with large errors because only two measurements were made per subject

# indicate a problem with low power, and that at least one of these low

# p-values is a type I error. Since there does not appear to be a relationship

# between the mean and variance, the variances can be pooled in order to

# increase power.

# Test whether the pooled variances are different.

# As above, var.test() can be used for this, but it isn't entirely intuitive.

# You fit models for each method with subject as the predictor. The MSE of

# these models is the pooled variance of the methods. var.test() can accept

# the model objects for comparison.

small_lm = lm(value ~ 0 + as.factor(subject), small_pefr)

large_lm = lm(value ~ 0 + as.factor(subject), large_pefr)

summary(small_lm) # The residual standard error given is the standard deviation of method measurements.

summary(large_lm)

var.test(small_lm, large_lm) # This will properly perform a two-tailed test of the MSE of the two models.

# There's no indication that the pooled variances are different.

# Below is the longer way, which for some may be more intuitive than

# var.test(), and it also allows one to calculate confidence limits for

# plotting.

# Calculate pooled variances and degrees of freedom.

var_large = with(large_stats, sum(var * df) / sum(df))

large_pooled_df = sum(large_stats$df)

var_small = with(small_stats, sum(var * df) / sum(df))

small_pooled_df = sum(small_stats$df)

# Calculate the p-value.

F_0 = var_small / var_large

print(pf(F_0, small_pooled_df, large_pooled_df, lower=FALSE) + pf(1/F_0, large_pooled_df, small_pooled_df)) # The same p-value as from var.test() above.

# Get confidence limits from distributions.

small_lower = var_small * small_pooled_df / qchisq(1 - 0.05 / 2, small_pooled_df)

small_upper = var_small * small_pooled_df / qchisq(0.05 / 2, small_pooled_df)

large_lower = var_large * large_pooled_df / qchisq(1 - 0.05 / 2, large_pooled_df)

large_upper = var_large * large_pooled_df / qchisq(0.05 / 2, large_pooled_df)

methods = factor(c('Mini-Wright', 'Wright'), levels=c('Mini-Wright', 'Wright'))

png(filename='PEFR pooled variance plot.png', height=80, width=55, res=600, units='mm', pointsize='10')

par(tck=0.02, mar=c(3.75, 2.25, 0.25, 0.25))

# Make an empty plot with the correct scale.

plot(c(100, 200) ~ methods,

main=NULL,

col=c('orange', 'blue'),

ylim=range(pretty(sqrt(c(0, small_lower, small_upper, large_lower, large_upper)))),

yaxt='n',

ylab='',

xlab=NULL,

pch=21,

bg='white')

arrows(1, sqrt(small_upper), 1, sqrt(small_lower), length=0.1, angle=90, code=3, col='orange')

arrows(2, sqrt(large_upper), 2, sqrt(large_lower), length=0.1, angle=90, code=3, col='blue')

points(sqrt(c(var_small, var_large)) ~ methods, col=c('orange', 'blue'), pch=21, bg='white')

title(ylab='Within-subject standard deviation (l/min)', line=1.25)

axis(2, at=seq(0, 30, by=10), label=rep("", 4))

axis(2, at=seq(0, 30, by=10), tck=0, lwd=0, line=-0.7)

dev.off()
