# Supplementary material for: To have value, comparisons of high-throughput phenotyping methods need statistical tests of bias and variance
Source: Front Plant Sci. 2024 Jan 19;14:1325221. doi: 10.3389/fpls.2023.1325221 (PMC10835710; doi:10.3389/fpls.2023.1325221)
Supplement: Supplementary file 6 [file Presentation_1.pdf]

Supplemental material

Section S1: A description of how *r* is not appropriate for method comparison

As an example of how *r* provides no means to compare the quality of two methods, assume there is an experiment to compare an established approach, method A, to measure crop height and a new approach, method B. In the experiment, each method was used to measure heights of several canopies, and *r* for their correlation was 0.6. Not being particularly high, one might conclude that method B is not good. The logical inconsistency with this conclusion is that, in itself, *r* indicates only whether two measures agree with each other. To conclude that method B is bad requires the implicit assumption that there is something special regarding method A; that it is in fact a measure of truth and that poor agreement with method A means that method B is poor. In some cases, this may be logical, but for this experiment, it is not. Assuming that method A is the truth presumes something about the very thing the experiment is meant to test. In reality, the methods could fail to agree, and thus have a small *r*, when method A is a poor measure of height, and method B an excellent measure, or both methods could be bad in different ways or both be good but not be linearly related. A small *r*, indicates only that two methods disagree, not which is better.

Furthermore, since *r* depends on the experimental design, a potentially worse outcome is to incorrectly conclude that because *r* is large the new method is equivalent to or better than the old one. For example, assume that methods A and B are both perfectly accurate, but the new method, B, has lower precision. If two experiments are conducted with these methods where one experiment has heights between 0.1 m and 2 m, and the second experiment has heights between 0.1 m and 4 m, *r* will be larger in the second experiment than the first, despite the same methods being used in both experiments. This can be extended and in fact, for any methods that are linearly correlated to any degree, *r* can be made arbitrarily close to unity simply by increasing the range of measured values, regardless of the relative accuracy or precision of either method. Because of this, it is possible to devise an experiment in which a new, poorer, method can produce a high correlation with an established, better, method. With such an experiment, by using *r* one would make the incorrect conclusion that the new method is equivalent to or better than the old one.

Section S2: The statistical model for bias and precision

For explanation, assume there is a plot for which one wants to know the crop height. The following terms can be defined:

μ: the true crop height of the plot.

$y_i^M = \mu + b^M + e_i^M$: measurements of the crop height using some method M, where *i* ranges from 1 to *n*, the number of observations, and e_i^M is a random, independent error drawn from some distribution, e^M, that has a mean of 0 and a finite variance of σ_M^2.

The measurement of the crop height differs from the true value on average by some amount b^M, the bias, and there is noise for any individual measurement due to the error term, e_i^M. Bias is a measure of accuracy and can be estimated as the difference between the mean of several measurements and the true value: $\hat{b}^M = mean(y_i^M) - \mu$ (a hat over the variable name is used here to indicate an estimate). However, the true value is most often unknown. In place of known true values, references are sometimes used, but again in some cases, such as with the height of a crop, there is no sensible reference. In these cases, bias from the true value cannot be determined, but one can calculate bias between methods as the difference between the means of repeated measurements of each method: $\hat{b}^{AB} = mean(y_i^A) - mean(y_i^B)$. Precision can be quantified as the variance of the measurements, which is an estimate of the variance of e^M: $\hat{\sigma}_M^2 = var(y_i^M)$.

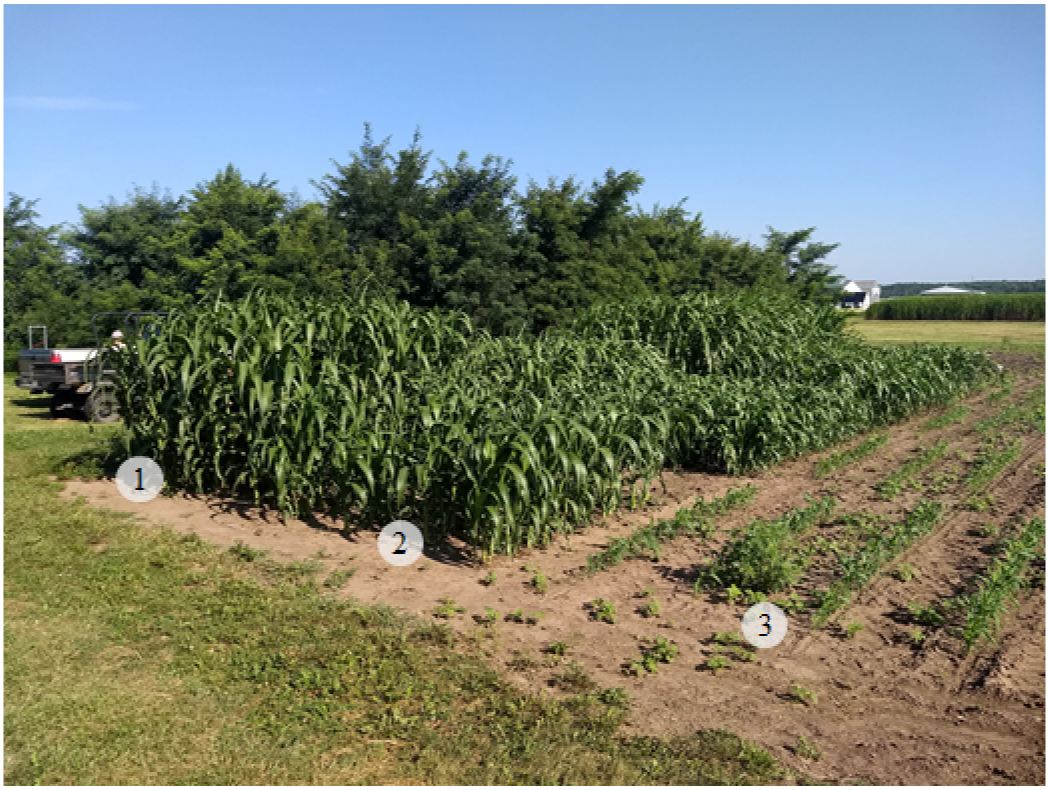

Supplemental Figure 1 shows the 2018 experimental plots at the Energy Farm (Urbana, IL). There were four sorghum varieties planted on three dates in 2018 (numbers 1, 2 and 3). The first date (number 1) was planted June 7. The same four varieties were planted on June 25 (number 2) and again on July 19 (number 3). This design allowed for measurements of crops at different growth stages within a single day.

Year	Number of varieties	Planting dates (1,2,3) (M-DD)	Measurement date	k (number of plots)	n^{Lidar} (number of measurements)	$n^{Tape\ Measure}$ (number of measurements)	$n^{Licor\ 2200}$ (number of measurements)
2018	4	(6-7, 6-25, 7-19)	8-31	12	5	7	5
2019	1	(5-31, 6-18, 7-15)	8-28	3	5	5	5
2020	3	(6-16, 7-16, 8-16)	9-18	9	5	5	5

Supplemental Table S1. This table catalogs the number of energy sorghum varieties, planting dates (M-DD), number of plots and number of measurements in each plot for each method (n^{Lidar}, $n^{Tape\ Measure}$, and $n^{Licor\ 2200}$).
